# Microbial and functional diversity of *Cyclopia intermedia* rhizosphere microbiome revealed by analysis of shotgun metagenomics sequence data

**DOI:** 10.1016/j.dib.2020.106288

**Published:** 2020-09-07

**Authors:** Ahmed Idris Hassen, Rian Pierneef, Z.H. Swanevelder, F.L. Bopape

**Affiliations:** a Agricultural Research Council, Plant Health and Protection, Private Bag X134, Queenswood, Pretoria 0121, South Africa; b Agricultural Research Council, Biotechnology Platform, Private Bag X05, Onderstepoort 0110, South Africa

**Keywords:** Cyclopia, Metagenomics, Microbial diversity, Rhizosphere, Proteobacteria, Annotation

## Abstract

*Cyclopia* spp., commonly referred to as *honeybush* due to the honey scented flowers, are indigenous legumes mainly growing in the Cape Floristic Region of the Western Cape, South Africa. Dozens of species, including *Cyclopia intermedia, C. subternata, C. plicata, C. genistoides* are used to make the well-known, popular and widely enjoyed beverage called ‘*honeybush tea’*. In the past, most rhizosphere microbial studies associated with *Cyclopia* spp. focused mainly on the taxonomy and diversity of the root nodule associated symbiotic nitrogen fixing rhizobia. The work presented here is the first report on the microbial and functional diversity of rhizosphere microbiome associated with *Cyclopia intermedia*. Metagenomic shotgun sequencing was performed on the rhizosphere soil sample collected from this *Cyclopia* sp. using illumina Hiseq 2500 platform which resulted in an α- diversity of 312 species. Analysis of the metagenome sequence using the Metagenomic analysis server (MG-RAST) indicated that bacteria constitute the dominant domain followed by Eukaryota, Archaea and other sequences derived from fungi and viruses. Functional diversity of the metagenome based on analysis using the Cluster Orthologous Group (COG) method showed metabolism as the most important function in the community. The raw sequence data is uploaded in FASTQ format on MG-RAST server with ID mgm4855911.3 which can be accessed at http://www.mg-rast.org/linkin.cgi?project=mgp90368. The data on the microbial and functional diversity of the rhizosphere community of *Cyclopia intermedia* generates a baseline information about the microbial ecology of this indigenous legume. The microbial profile data can also be used as indicators of soil health characteristic of the rhizosphere of this important legume.

## Specification Table

**Table:** 

Subject	Microbiology
Specific subject	Microbial Metagenomics
Type of Data	Figures, Charts Assembled DNA sequence
How data were acquired	Data were generated using HiSeq 2500 paired-end 125 bp sequencing with the SBS V4 Kit chemistry of Illumina.
Data format	Raw data in FASTQ format
Parameters of data collection	Soil samples associated with the rhizosphere of *Cyclopia intermedia* were collected from farmers’ fields with permission
Data Source location	Agricultural Research Council, Pretoria, South Africa Sample collection site: Haarlem, Western Cape, South Africa (GPS 33°44′02″S 23°20′19″E)
Description of data collection	Soil samples from the rhizosphere of *Cyclopia intermedia* were collected and stored in sterile bags. All samples were transported on ice and stored at 4 °C until processing. DNA was extracted from 250 mg sub-soil sample with the ZymBIOMICS DNA/RNA kit according to the protocol. DNA was subsequently fragmented with a Covaris into 350 bp sizes prior to library preparation with the TruSeq Nano DNA low throughput preparation kit (Illumina).
Data accessibility	The data is uploaded at MG-RAST server with ID: mgm4855911.3 and can be accessed at. https://www.mg-rast.org/linkin.cgi?project=mgp90368

## Value of the Data

• This data provides vital information on the microbial and functional diversity of the rhizosphere community of the economically important *Cyclopia intermedia*. Understanding the microbial and functional diversity of a given soil is useful in sustainable agriculture as it can be used as a microbial indicator of soil health.
• This data generates useful information for researchers and students involved in soil microbiology and microbial ecology of *Cyclopia* spp.
• The data also provides baseline information on the abundance of the rhizobia complex (*Rhizobium, Bradyrhizobium, Sinorhizobium,* and *Mesorhizobium* spp. for further screening of these group of bacteria as potential nitrogen fixers (biofertilizers) in honeybush cultivation.

## 1 Data Description

**Fig. 1:**
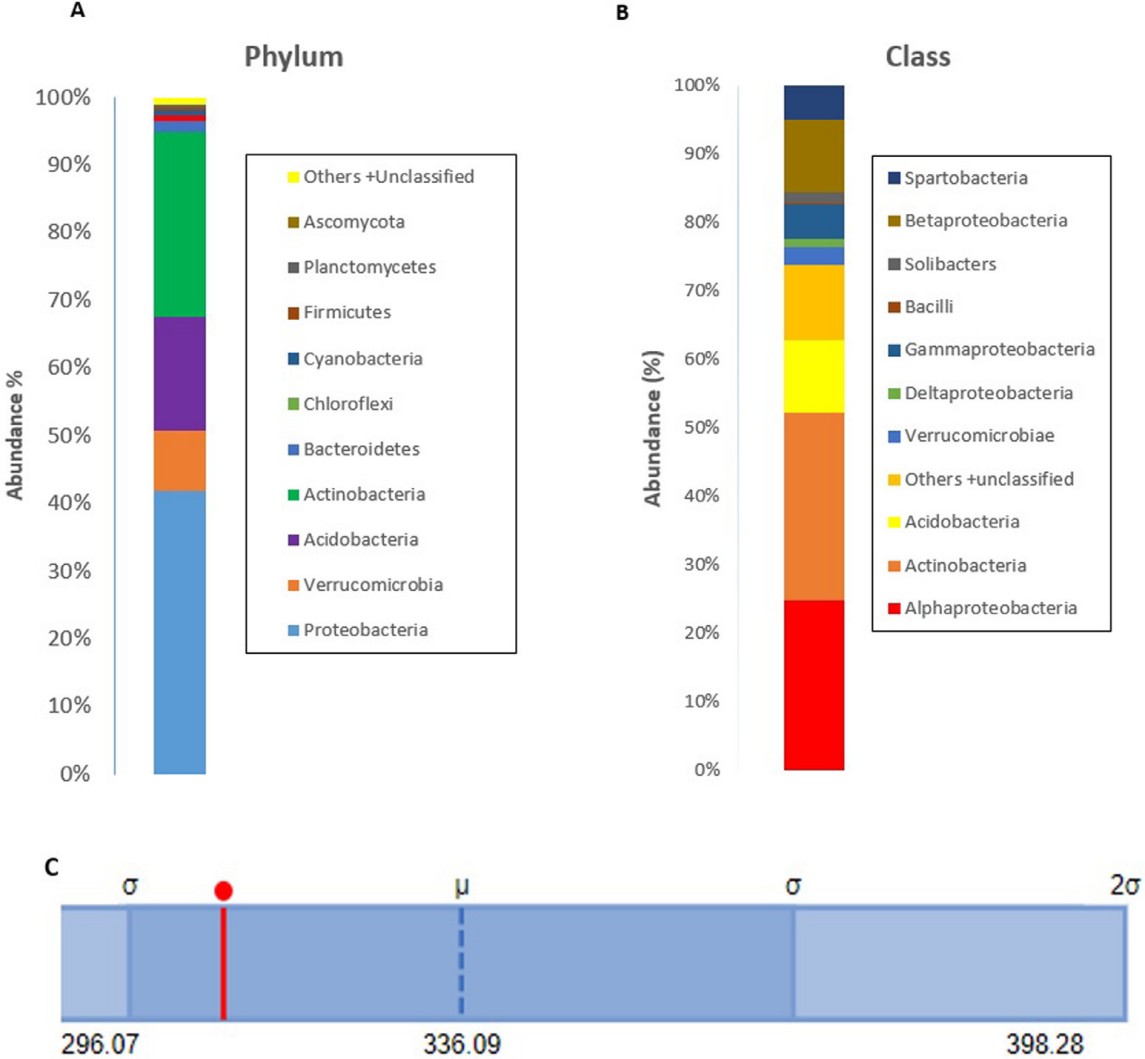
Taxonomic abundance and α-diversity of the microbiome of *C. intermedia* rhizosphere after analysis of the metagenome sequence on MG-RAST. **A**. Abundance at phylum level using stacked column shows Proteobacteria as the most abundant taxon (41.82%) folllowed by Actinobacteria (27.26%) and Acidobacteria (16.77%). **B**. Abundance at class level was higher for Actinobacteria (27.6%), Alphaproteobacteria (24.9%) and Betaprotoebacteria (10.47). Taxa with abundance level < 1% are combined and presented as others + unclassified derived from viruses and fungi. **C**. α-diversity summarizing the diversity of organisms in the analysed metagenome. The figure shows the minimum, maximum and mean values together with the standard deviation ranges (σ and 2σ) in light shades. The α-diversity, which is 312 species, is indicated in red.

**Fig. 2:**
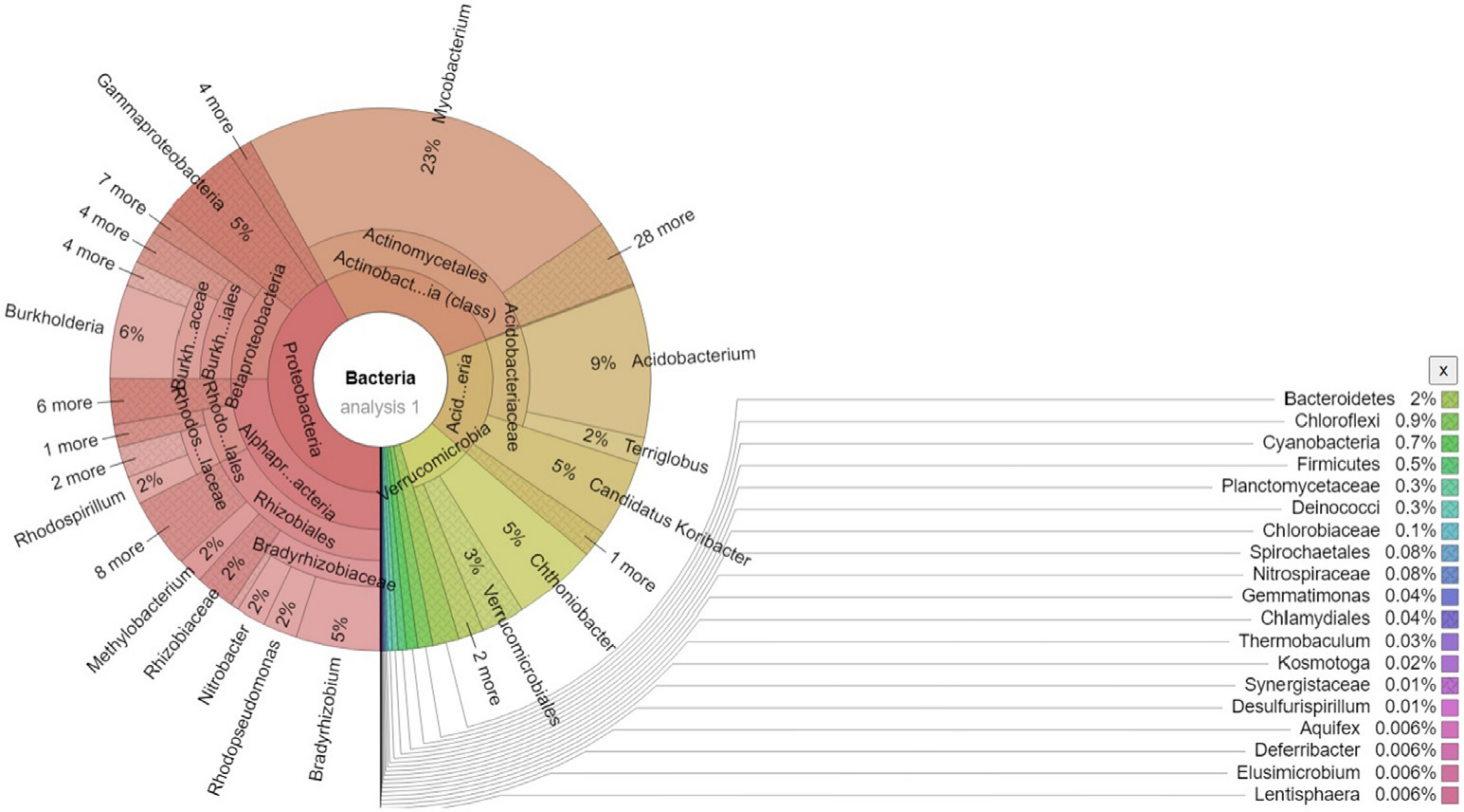
Krona chart for taxon abundance (%) at the genus level of the dominant domain (bacteria) analyzed by MG-RAST using a modification of the default parameters: *E* = 10,% identity = 75% and minimum alignment length = 100. The most dominant genus is *Mycobacterium* (23%), followed by *Acidobacterium* (9%), *Burkholderia* (6%), *Candidatus Koribacter, Chtoniobacter* and *Bradyrhizobium* each at (5%).

The community metagenome sequence has a total of 58, 320, 141 bp read length producing 72,096 contigs with a mean sequence length of 809±703 and mean GC content of 55±17%. There are 57,880 identified protein features and 134 identified rRNAs. Analysis of the metagenome sequence for taxon abundance at domain level indicated that the dominant taxa belong to bacteria (99.58%) followed by Eukaryota (0.32%), Archaea (0.09%) and other sequences derived from fungi and viruses (0.01%). At Phylum level, the most dominant taxa in descending order include Proteobacteria, Actinobacteria and Acidobacteria whereas the taxon abundance at class level was higher for Actinobacteria followed by Alphaproteobacteria, Acidobacteria and Betaproteobacteria. Further analysis of the taxon abundance of the predominant domain indicated that the top most abundant genera include *Mycobacteriaum* (23%) followed by *Acidobacteria* (9.04%), *Burkholderia* (5.59%) and *Bradyrhizobium* (5.04%). The details of the taxonomic abundance at phylum, class and genus level are presented in (Figs. 1& 2). *Cyclopia intermedia* is one of the legumes used in the production of the local beverage tea both for home consumption and commercialization. Due to this, the taxonomic abundance of selected symbiotic nitrogen fixing bacteria as well as some known beneficial rhizobacteria commonly found in the rhizosphere of several crops is of particular importance and therefore an extract from the annotated data is presented in Table 1. The α-diversity of the annotated metagenome i.e. summary of the diversity of the total number of organisms is estimated from the distribution of the species level abundance and contains 312 species (Fig. 1C).

**Table 1:** Taxonomic abundance (%) at the genus level for selected common symbiotic nitrogen fixing and free living bacteria within the microbiome of *C. intermedia* rhizosphere. The list is in descending order starting with the most abundant genus and most number of hits at (*E* *=* *10,% ID = 75, alignment length = 100*)*.

Genus	Abundance (%)	Number of Hits	Number of Species	Abundance(%) within Proteobacteria
Burkholderia	6.0	883	21	13
Bradyrhizobium	5.0	796	3	12
Rhizobium	1.0	178	2	3
Pseudomonas	0.9	142	11(14)**	2
Cupriavidus	0.9	136	4	2
Azosprillum	0.83	131	1	2
Mesorhizobium	0.8	129	2	2
Sinorhizobium	0.8	120	3	2
Bacillus	0.08	13	8	2
Azotobacter	0.06	9	1(22)**	1

* Analysis parameters where *E*= the number of similar scoring alignments one expects to see by chance in the database searched;*% ID* = sequence similarity. **Number of species (in the parenthesis) for *Pseudomonas* and *Bacillus* are higher when analyzed with the default parameters (*E* *=* *5,%ID = 60, alignment length = 15*), whereas for the rest of the genera the analysis gave the same result using both the default and changed parameter.

**Fig. 3:**
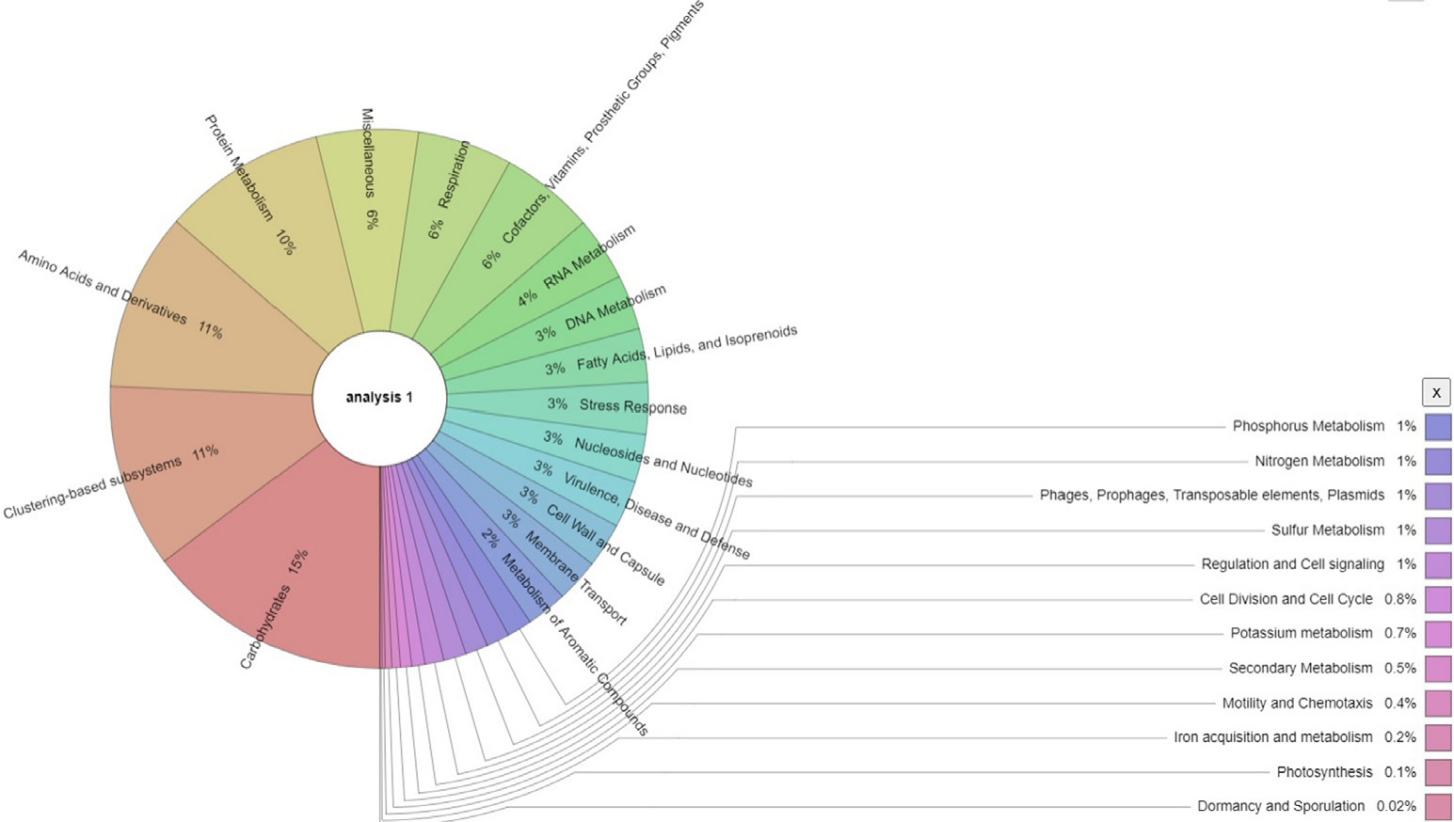
Krona chart showing functional hierarchical profiles of the metagenome of *C. intermedia* rhizosphere analyzed using the SEED Subsystem high level annotation. Note that carbohydrate metabolism is the dominant function (15%) followed by amino acids and derivatives (11%), clustering based systems (11%) and protein metabolism (10%).

Analysis of the metagenome for major functional profiles indicated that metabolism was the dominant feature (53.4%) followed by cellular processes and signaling (16.8%), information storage and processing (16.5%) and poorly characterized features at 13.5%. Analysis using the SEED Subsystem annotation at higher level showed carbohydrate metabolism being the highest with 15% followed by aminoacids and derivatives (11%) and protein metabolism (10%). Other essential nutrient metabolisms like Nitrogen (N), Phosphorous (P) and Sulphur (S) metabolisms have all 1% abundance (Fig. 3). Taxonomic abundance at the genus level as well as functional diversity of the entire metagenome are indicated using the Krona interactive web tool [5] (Figs. 2, 3). Additional information on all the data is available with the submitted supplementary materials. The entire raw read sequence files were deposited in FASTQ format at the MG-RAST server and are publicly accessible at (https://www.mg-rast.org/linkin.cgi?project=mgp90368).

## 2 Experimental Design, Materials and Methods

### 2.1 Sample collection

Soils samples were collected from the rhizosphere of *Cyclopia intermedia* (honeybush) at five different sites of a private farm in Haarlem, Western Cape, South Africa (GPS 33°44′02″S 23°20′19″E) with permission. On the field the honeybush roots penetrate deeper than 8 inches into the rhizosphere. Thus, approximately 1 kg of the top 6 to 8 inches of the rhizosphere soil was removed and transferred into sterile plastic bags prior to cooling in cooler boxes with ice blocks and transportation to the laboratory. The soil types in the rhizosphere of *Cyclopia intermedia* at all five sites of the Haarlem area are darkly colored loamy soils. A total of 25 samples were collected from five sites, each site containing five samples. The five samples from the same sites were first pooled and finally from each subset of pooled sample, 20 g was collected to make 100 g pooled sample.

### 2.2 DNA extraction and metagenomics shotgun sequencing

Microbial community DNA was extracted from a 250 mg subsample taken randomly from the pooled *C. intermedia* rhizosphere soil samples and extracted with ZymBIOMICS DNA/RNA extraction kit in accordance with the manufacturer's instruction. DNA was subsequently fragmented with a Covaris sonicator into 350 bp sizes prior to library preparation with the TruSeq Nano DNA low throughput preparation kit (Illumina) and sequencing. Shotgun metagenomics sequencing was done using a HiSeq 2500 (Illumina) using the paired-end 125 bp SBS V4 Kit chemistry (Illumina).

### 2.3 Bioinformatics analysis of sequence data

Raw reads were quality controlled and filtered using Trimmomatic version 0.36 [1]. Spades version 3.12 [2] in which the “meta” parameter specified was used to construct the metagenomic assembly. Annotation and analysis of the data were performed using Metagenomics-Rapid Annotation Subsystem Technology (MG-RAST) version 4.3 [3].For taxonomic analysis, the data sets were processed by aligning the contigs against the RefSeq protein database using a modification of the default parameter (E-value = 1 × 10^−10^,% identity 75% and minimal alignment length of 100). Whereas functional profile analysis was made using the Cluster of Orthologous Group (COG) at higher level category (level 1) to predict the number of contigs with predicted functions [4].

## Declaration of Competing Interest

The authors hereby declare that we have no known conflict of interests that might affect the work reported in this article.

## Appendix Supplementary materials

Image, application 1

Image, application 2

Image, application 3

Image, application 4

Image, application 5
